# Abnormal glucose tolerance and insulin resistance in polycystic ovary syndrome amongst the Taiwanese population- not correlated with insulin receptor substrate-1 Gly972Arg/Ala513Pro polymorphism

**DOI:** 10.1186/1471-2350-7-36

**Published:** 2006-04-07

**Authors:** Ta-Chin Lin, Jui-Mei Yen, Kum-Bing Gong, Tsung-Cheng Kuo, Dong-Chi Ku, Shu-Fen Liang, Ming-Jiuan Wu

**Affiliations:** 1 Department of Gynecology, Obstetrics, and Infertility, Kuo General Hospital, No. 22, Section 2, Ming-Sheng Road, Tainan, 70054, Taiwan; 2 Department of Pediatrics, SinLau Christian Hospital, No. 57, Section 1, Eastgate Road, Tainan, 70142, Taiwan; 3 Department of Food Health, Chia-Nan University of Pharmacy and Science, 60 Erh-Jen Road, Section 1, Jen Te, Tainan, 71710, Taiwan

## Abstract

**Background:**

Insulin resistance and glucose dysmetabolism in polycystic ovary syndrome (PCOS) are related with the polymorphisms in the genes encoding the insulin receptor substrate (IRS) proteins, especially Gly972Arg/Ala513Pro polymorphism being reported to be associated with type-2 diabetes and PCOS. We intended to assess the prevalence of abnormal glucose tolerance (AGT) and insulin resistance in Taiwanese PCOS women. We also tried to assess whether the particular identity of Gly972Arg/Ala513Pro polymorphic alleles of the IRS-1 gene mutation can be used as an appropriate diagnostic indicator for PCOS.

**Methods:**

We designed a prospective clinical study. Forty-seven Taiwanese Hoklo and Hakka women, diagnosed with PCOS were enrolled in this study as were forty-five healthy Hoklo and Hakka women as the control group. Insulin resistance was evaluated with fasting insulin, fasting glucose/insulin ratio, and homeostasis model assessment index for insulin resistance (HOMA_IR_). The genomic DNA of the subjects was amplified by PCR and digested by restriction fragmented length polymorphism (RFLP) with *Bst N1 *used for codon 972 and *Dra III *for codon 513.

**Results:**

AGT was found in 46.8% of these PCOS patients and was significantly related to high insulin resistance rather than the low insulin resistance. Those patients with either insulin resistance or AGT comprised the majority of PCOS affected patients (AGT + fasting insulin ≥17: 83%, AGT + glucose/insulin ratio ≥6.5: 85.1%, AGT + HOMA_IR _≥ 2: 87.2%, and AGT + HOMA_IR _≥ 3.8: 72.3%). None of the tested samples revealed any polymorphism due to the absence of any *Dra III *recognition site or any *Bst N1 *recognition site in the amplified PCR fragment digested by restriction fragmented length polymorphism.

**Conclusion:**

There is significantly high prevalence of AGT and insulin resistance in PCOS women, but Gly972Arg and Ala513Pro polymorphic alleles of IRS-1 are rare and are not associated with the elevated risk of PCOS amongst Taiwanese subjects. This is quite different from the similar study in phylogenetically diverged Caucasian subjects.

## Background

Polycystic ovary syndrome (PCOS) is one of the most-common endocrine disorders for premenopausal women with a prevalence rate of 4–12% internationally [1]. PCOS is characterized by irregular menses, chronic anovulation, infertility, hyperandrogenism, and insulin resistance [2]. In 1991, Poretsky and Nestler found insulin to be an effector of ovarian and adrenal steroid metabolism, and postulated the paradox of insulin-induced hyperandrogenism within insulin-resistant states [3]. In addition to being involved as a predisposing factor for type-2 diabetes, insulin resistance plays a key role in the pathogenesis of PCOS [4].

Insulin receptor substrate (IRS) proteins are critical to signal transduction in insulin target tissues [5]. The binding of insulin to its receptor induces the phosphorylation of the cytosolic substrates IRS-1 and IRS-2 [6]. Nevertheless, the biokinetic response of IRS-1 and IRS-2 to tyrosine protein kinases depends upon the binding specificity and affinity of the tyrosine phosphorylation sites within the IRS, which can be altered by mutated aminoacid polymorphisms within the phosphotyrosine-binding (PTB) domain [7]. Among these identified tyrosine phosphorylation sites. [6,7], mutations of Gly972Arg, Pro170Arg and Met209Thr have revealed different level of reduction in Phosphatidylinostiol 3-kinase (PI 3-kinase) activity [8]. The impaired insulin-signaling pathway for PI 3-kinase activity plays a role in the development of insulin resistance [7-9]. Therefore, the Ser892Gly and Thr608Arg polymorphisms for non-insulin dependent diabetes mellitus (NIDDM) patients were reported, such a mutation revealing a consequent decrease in insulin-induced phosphorylation and PI 3-kinase activity [10,11].

Beside influencing an individual's susceptibility to NIDDM, these polymorphisms have been shown to be associated with phenotypic features of PCOS [12,13]. We chose the Gly972Arg and Ala513Pro variants of the IRS-1 gene for investigation, because these specific allelic variants are located near the Tyr-Met-X-Met (YMXM) motifs around Tyr987 and Tyr612 and because these variants have been reported to influence insulin resistance, hyperinsulinemia and fatty-acid composition of muscles with a non-sporadic prevalence [7,10,14]. Our purpose was to identify, within the Taiwanese population of PCOS, the prevalence of the IRS-1 gene mutation with impaired tyrosine kinase activity, in order toassess whether the identity of the IRS-1 gene mutation of codon 513 (GCC->CCC) and codon 972 (GGG->AGG) can act as an appropriate diagnostic indicator for Taiwanese PCOS characterized by hyperinsulinemia and hyperandrogenism.

There were apparently racial differences in the phenotypic expression of insulin resistance as well as the genetic mutations in different races with PCOS [15,16]. Because of the racial differences in the phenotypic and genotypic expression of PCOS-affected subjects, we purposely focused our study on the genotypic polymorphism in so-called" Taiwanese"habitants in Taiwan [17]. The population of Taiwan area were composed of Taiwnanese Hoklo (or Minnan), Taiwanese Hakka (or Haka), immigrant Chinese mainlanders, and aborigines [17,18]. In traditional term, so-called" Taiwanese" are Taiwanese Hoklo and Hakka. We focus our study on the Taiwanese Hoklo and Hakka subjects, because that the genotypes of the immigrated Chinese mainlanders were greatly diversed and the aborigines as Malayo-Polynesians were even more different from Han Chinese people [17,18].

## Methods

### Subjects

The presence of PCOS was defined according to criteria arising from a National Institutes of Health (NIH) in 1990 and its later modification. A diagnosis of PCOS was made according to the criteria defined by: (i) hyperandrogenism, (ii) oligo-ovulation, and (iii) the exclusion of related disorders (see below). Another diagnosis of PCOS was also made, when the hyperandrogenism can not be definitely proved, with the criteria defined by: (i) oligo-ovulation (ii) increased LH/FSH (luteinizing hormone/follicle stimulating hormone) ratio >2 (iii) specific criteria for PCOS in an ultrasound scan (see below) [19]. These criteria are also adapted and included in newly proposed criteria by 2003 ASRM/ESHRE Rotterdam definition.

In the criteria of hyperandrogenism, biochemical hyperandrogenism was defined as a serum total testosterone level > 0.8 ng/ml or testosterone > 0.7 ng/ml with sex hormone binding globulin (SHBG) < 30 nmol/L [16]. The ultrasound definition of polycystic ovary was defined as follows: "increased ovarian area (>5.5 cm^2^/ovary) or volume (>11 m^3^/ovary) and/or presence of ≥12 follicles measuring 2–9 mm in diameter (mean of both ovaries)"[20,21]. The following diseases were excluded from our study: hyperthyroidism, hypothyroidism, congenital adrenal hyperplasia (abnormal 17-hydroxyprogesterone level), pituitary insufficiency, pituitary tumor, and pre-diagnosed known diabetes mellitus. Additional to NIH 1990 criteria of PCOS, Rotterdam 2003 definition of PCOS expended two additional phenotypes, some of them were excluded because that these criteria increase the phenotypic heterogeneity of the disorder and their use will likely decrease the ability of genetic and other molecular studies [19].

From November 2002 to July 2005 inclusively, forty-seven subjects, of which thirty-seven were Taiwanese Hoklo people (78.7%), ten subjects were Taiwanese Hakka (21.3%), and no aboriginal or mainlanders, were recruited for this study from the infertility/endocrine/obesity clinics of the Kuo General Hospital, according to the above criteria. Another fifty patients were enrolled from the normal population without clinical PCOS to constitute the primary control group. In the further biochemical and sonographic evaluation, five subjects were excluded due to abnormal biochemical androgen level, LH/FSH ratio ≥ 2, or abnornal ovarian sonographic finding. The residual forty-five patients, thirty-eight Taiwanese Hoklo individuals (84.4%) and seven Taiwanese Hakka (15.6%), constituted the final control group. This study has been approved by the Kuo General Hospital Ethics Committee.

### Laboratory tests

Fasting insulin, fasting glucose, testosterone, SHBG, FSH, LH, and prolactin levels were all determined. Plasma levels of FSH, LH, prolactin, and SHBG were determined using immunofluorometric assays, and the testosterone and insulin levels were determined using radioimmunoassay study.

The abnormal glucose tolerance (AGT) was defined as either impaired glucose tolerance or diabetes mellitus in the 75 gram oral glucose tolerance test (OGTT) by American Diabetes Association criteria. The level of insulin resistance was evaluated with respect to fasting insulin level, the glucose:insulin ratio [glucose (mg/dl)/insulin (uU/ml)], and the HOMA_IR_index (homeostasis model assessment index for insulin resistance) [fasting insulin (uU/ml) × fasting glucose (mg/dl)/405].

### Extraction of genomic DNA

Genomic DNA was extracted from peripheral blood mononuclear cells by using a QIAamp DNA Blood Mini Kit (QIAGEN GmbH, Hilden, Germany). The purity, size and concentration of the isolated DNA was measured by A260/A280 and agarose gel electrophoresis. PCR was used to amplify two regions of the franking codon 972 and codon 513 of IRS-1.

### PCR and Restriction fragmented length polymorphism (RFLP)

According to the DNA sequences proposed in 1995 by Hitman et al. [22], the sequences of the primers for PCR are listed as: (1) The primers for Codon 513: Forward primer, 1953 5'GCG GTG AGG AGG AGC TAA GC 3' 1972, and Reverse primer 2200 3' GGG CAG GGT CAG GAG TCA CCG 5' 2220; (2) The primers for Codon 972: Forward primer, 3339 5' CTT CTG TCA GGT GTC CAT CC 3' 3358, and Reverse primer 3582 3' CGA TGC ACC TGT GGA GCG GT 5' 3601.

PCR was performed using 50 ul primers with 0.3 ug of genomic DNA. The assay conditions were: 10 mmol/L Tris-HCl (pH = 9.0), 50 mmol/L KCl, 1.5 mmol/L MgCl_2_, 0.1% Triton X-100, and 0.2 mmol/L of the four deoxynucleoside triphosphates (Promega, Madison, Wisconsin, USA). The selected samples were subjected to 35 cycles of amplification: denaturation at 94°C for one minute, annealing at 60°C for one minute, and extension at 72°C for two minutes. The resulting products were 268 bp and 263 bp for codon 513 and 972, respectively.

The PCR products were digested with restriction enzyme(s) followed by agarose gel electrophoresis: 1) For the PCR products of codon 972 digested with *Bst N1*, the resulting wild-type samples contained three bands sized 23, 81 and 159 bp and the mutant samples contained four bands sized 23, 81, 108 and 51 bp. We noted five bands sized 23, 81, 159, 108 and 51 bp for Gly972/Arg972 heterozygotes and four bands sized 23, 81, 108 and 51 bp for Arg972 homozygotes. 2) For the PCR products of codon 513 digested with *Dra III*, the resulting wild-type samples contained one band sized 268 bp, and the mutant samples contained two bands sized 168 bp and 100 bp; three bands sized 268 bp, 168 bp and 100 bp were noted for Ala513/Pro513 heterozygotes and two bands sized 168 bp and 100 bp for Pro513 homozygotes.

### Statistical analysis

Data were analysed by using the Statistical Program Social Sciences (SPSS 10.0.7C for Windows). Results were presented as mean values ± standard deviation (SD). Comparisons between PCOS patients and normal control group were performed using Student's *t*-test. It was also used to compare the variables in abnormal and normal glucose tolerance patients. A value of *P *< 0.05 was considered statistically significant.

## Results

### Characteristics of the 47 patients with PCOS

Forty-seven women were finally included in the diagnosis of PCOS. Their average age was 26.3 ± 5.4 years ranging from 17 to 36 years old. They also had higher body weight and body mass index (BMI) (average weight: 72.4 ± 14.5 kg; range, average BMI: 28.5 ± 6.0) than the normal population (average age 24.6 ± 3.9 years, average weight: 56.4 ± 9.8 kg, average BMI: 21.7 ± 3.2). The clinical feature distributions of our patients are shown in Table 1. Thirty-one (65.9%) of the 47 women were obese (BMI ± 25) and fifteen (31.9%) had acanthosis nigrans.

**Table 1:** Clinical and laboratory features of 47 patients suffering from PCOS and 45 controls included in this genetic study

Characteristics	PCOS (n = 47)	Normal (n = 45)	*P*
Age (years)	26.3 ± 5.4	24.6 ± 3.9	NS
Acanthosis nigrans (%)	31.9%	0%	<0.05
Body weight (kg)	72.4 ± 14.5	56.4 ± 9.8	<0.05
BMI (kg/m^2^)	28.5 ± 6.0	21.7 ± 3.2	<0.05
LH/FSH	2.1 ± 1.0	1.3 ± 0.6	<0.05
Prolactin (ng/ml)	18.6 ± 8.0	12.7 ± 6.3	NS
Testosterone (ng/ml)	1.3 ± 0.5	0.5 ± 0.2	<0.05
SHBG	21.1 ± 8.3	48.1 ± 7.8	<0.05
Fasting glucose (mg/dl)	91.3 ± 18.1	86.3 ± 17.6	NS
IGT (%)	36.2%	6.25%	<0.05
AGT (%)	46.8%	6.25%	<0.05
Fasting insulin (uU/ml)	24.4 ± 17.2	7.9 ± 6.7	<0.05
Glucose/insulin ratio	5.27 ± 3.34	12.13 ± 4.94	<0.05
HOMA_IR_	5.21 ± 3.73	1.59 ± 0.65	<0.05

PCOS = polycystic ovary syndrome; BMI = body mass index; SHBG = sex hormone binding globulin; IGT = intolerance of 75 gm oral glucose tolerance test; Glucose/insulin ratio = fasting glucose (mg/dl)/fasting insulin (uU/ml); AGT = abnormal glucose tolerance; HOMA_IR_(Homeostasis model assessment index for insulin resistance) = fasting insulin (uU/ml) × fasting glucose (mg/dl)/405

In biochemical assays, 45 (95.7%) of the 47 women with PCOS were found to have a testosterone level ± 0.8 ng/ml, and 2 (4.3%) < 0.8 ng/ml, with an average testosterone level of 1.3 ± 0.5 ng/ml. An insulin level (average 24.4 ± 17.2 uU/ml) ± 17 was found in 32 (68.1%) of the 47 patients. The average fasting blood sugar (91.3 ± 18.1 mg/dl) was normal. A ratio (average: 5.27 ± 3.34) of glucose to insulin ± 6.5 was found in 35 (74.5%) of the 47 patients. Decreased sex hormone binding globulin (SHBG) was also noted with an average of 21.1 ± 8.3 nmol/L. The averages of LH/FSH ratio (2.1 ± 1.0) and prolactin level (18.6 ± 8.0 ng/ml) were significantly increased (Table 1).

### Co-expression of abnormal glucose tolerance and insulin resistance

With normal fasting glucose levels, these PCOS patients had decreased SHBG levels than the control (21.1 ± 8.3 vs. 48.1 ± 7.8 nmol/L, p < 0.05), the insulin levels higher (24.4 ± 17.2 vs. 7.9 ± 6.7 uU/ml, p < 0.05) and the homeostasis model assessment index for insulin resistance (HOMA_IR_) higher (5.21 ± 3.73 vs. 1.59 ± 0.65 units, p < 0.05) (Table 1).

Twenty-two (46.8%) of these PCOS patients had abnormal results in the oral glucose tolerance test (OGTT) by American Diabetes Association criteria. None had pre-diagnosed diabetes mellitus. In these twenty-two (46.8%) AGT patients, five met the criteria of diabetes mellitus in our OGTT and seventeen (36.2%) met the criteria of glucose intolerance (IGT). Fasting insulin level, glucose/insulin ratio, and HOMA_IR _are all significantly related to the presence of abnormal OGTT test (p < 0.05). A high insulin group with insulin level ≥17 uU/ml constituted 68.1% (32/47) of the PCOS patients. Co-existence of abnormal OGTT and high insulin was found in 68.2% (15/22) of AGT group and 46.9% (15/32) of high insulin group. A low glucose/insulin ratio group with glucose/insulin ratio ≥6.5 constituted 74.5% (35/47) of the PCOS affected patients. In this low glucose/insulin ratio group, co-existence of abnormal OGTT and low glucose/insulin ratio was found in 77.3% (17/22) of AGT group and 48.6% (17/35) of low glucose/insulin group.

Regarding HOMA_IR _as the criteria of insulin resistance, a moderate-high HOMA_IR _group with HOMA_IR _≥2 constituted 76.6 % (36/47) of the PCOS affected patients. Co-existence of abnormal OGTT and moderate-high HOMA_IR _was found in 77.3% (17/22) of AGT group and 47.2% (17/36) of this moderate-high HOMA_IR _group. In the higher standard for diagnosis of insulin resistance, high HOMA_IR _group with HOMA_IR _≥3.8 constituted 53.2 % (25/47) of the PCOS affected patients. Co-existence of abnormal OGTT and high HOMA_IR _was found in 59.1% (13/22) of AGT group and 52% (13/25) of this high HOMA_IR _group. Regarding these markers for insulin resistance, those patients with either insulin resistance or AGT comprise the majority of PCOSaffected patients (AGT + fasting insulin ≥17: 83%, AGT + glucose/insulin ratio ≥6.5: 85.1%, AGT + HOMA_IR _≥2: 87.2%, and AGT + HOMA_IR _≥3.8: 72.3%).

### Neither Ala513Pro nor Gly972Arg polymorphism found

**Figure 1:**
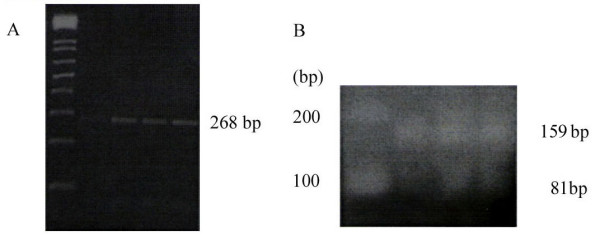
(A) The representative electrophoretic gel featuring the RFLP analysis of the codon 513 polymorphism in IRS-1. (B) The representative electrophoretic gel featuring the RFLP analysis of the codon 972 polymorphism in IRS-1.

In the PCR-RFLP analysis of the Ala513Pro polymorphism, all the tested samples from both PCOS and control groups revealed no polymorphisms because no *Dra III *recognition site was apparent for the amplified PCR fragment as represented in Figure 1A, all samples revealing a band of 268 bp.

In the PCR-RFLP analysis of the Gly972Arg polymorphism, none of the tested samples revealed any polymorphism as indicated in Figure 1B, all samples revealing a band of 159 bp rather than one of 108 bp plus one of 51 bp.

## Discussion

The incidence of the presence of a Gly972Arg variant has been reported to be greater for premature pubarche teenagers and hyperandrogenic adolescent girls featuring PCOS than for control women (respectively, 27.5%: 17% : 6.6%) [23]. Further, evidence has also supported the reporting of a gene-dosage effect by a Gly972Arg IRS-1 variant upon fasting insulin and HOMA values amongst PCOS-afflicted women [12]. The Gly972Arg IRS-1 variant has been reported tobe more prevalent amongst insulin-resistant PCOS patients compared with non-insulin-resistant PCOS patients or control subjects (respectively, 39.3 vs. 4.0 and 16.6%, p < 0.0031) [12]. Further, Ala513Pro variants have been previously detected for 3%–6.9% of NIDDM patients [24,25] and were found to be more prevalent for NIDDM patients who featured a high fasting insulin level than was the case for Gly972Arg variants [25]. Meanwhile, a high fasting insulin level is thought to be the initial state of hyperinsulinemia which is found frequently amongst PCOS-affected patients [1,16,26]. In our study, we evaluated the status of insulin resistance with fasting insulin, fasting glucose:insulin (G/I) ratio, and HOMA_IR_, all of which are parameters widely used to assess insulin resistance previously [1,12,15,27,28]. It was clearly apparent that there was a significantly greater incidence (72.3–87.2%) of either insulin resistance or AGT amongst our PCOS patients than was the case for the normal population, and the prevalence of either insulin resistance or AGT amongst our PCOS patients was certainly not less than that reported cases for PCOS subjects in other studies [12,15,26,29]. Nevertheless, as shown by our data, the higher prevalence of insulin resistance amongst our PCOS patients as compared to the controls is associated with a significantly greater rate of glucose intolerance, which correlates with the later development of NIDDM for PCOS subjects. The incidence (46.8%) of AGT in our study is similar to the incidence (45%) in the report of Ermann et al [29]. It seems higher than clinical expectation because that our subjects, enrolled from infertility/endocrine/obesity clinics, have higher body weigh and lower SHBG which are correlated with incidence of insulin resistance and AGT [16,30]. The population of PCOS is greatly different due to the collection source and criteria of PCOS. The patients enrolled from the infertility clinic seem to have lower body weight and higher SHBG than the patients enrolled from the endocrine/obesity. Meanwhile, another reason is that I do not include those multifollicularovaries (MFO), mimicing PCOS, due to other etiology, e.g. low BMI or adolescence. They may be included in 2003 ASRM/ESHRE Rotterdam definition of PCOS, but they could be excluded in our including criteria or NIH 1990 definition of PCOS.

According to the 2001 report of El Mkadem et al., the variants Gly972Arg of IRS-1 and Gly1057Asp of IRS-2 occurred more frequently for the PCOS population than was the case for the normal population [12]. Reportedly, the allelic frequency of the 972Arg variant of IRS-1 was 0.11, and the allelic frequency of the 1057Asp variant of IRS-2 was 0.36 for PCOS individuals, and the Gly972Arg IRS-1 variant for PCOS individuals featuring a high fasting insulin was ten-fold greater than it was for PCOS patients exhibiting a low fasting insulin and 2.3-fold greater than for control women. From the 2002 study of Ehrmann et al., however, no association of the IRS-1 Gly972Arg genotype with any clinical or hormonal measure was reported for nondiabetic PCOS subjects [26]. From our study, independently of whether patients featured NIDDM or not, the results also revealed no evidence of any polymorphism in the regions encoding codon 972 of the IRS-1 for the Taiwanese population, although 36.2% of our PCOS subjects revealed AGT and 72.3–87.2% revealed either insulin resistance or AGT with respect to different criteria of insulin resistance. It would appear that there may be a racial difference in the mutated sites of IRS-1 polymorphisms featuring the similar insulin resistance. Indeed, such a result also enhances the suspicion that the screening value of insulin resistance as a possible marker for PCOS would likely be population-specific. For instance, a G/I ratio of ≤ 7.2 has been suggested for insulin resistance amongst white women suffering PCOS [15], whereas a corresponding figure of ≤ 4.0 has been proffered elsewhere for Mexican American featuring PCOS [15], as also a figure of ≤ 6.5 for Taiwanese women with PCOS [16].

In a study of Japanese women suffering NIDDM, codon 972/513 polymorphisms were observed for 3.6%/0% of study subjects (respectively, codon 972/codon 513), compared to a corresponding control value of 4.5%/0% (respectively, codon 972/codon 513) there being no significant difference between NIDDM patients and controls in this regard [31]. In the present study, our results have not revealed any evidence suggesting polymorphism in the regions encoding codons 972 and 513 of IRS-1 amongst the Taiwanese population. From the study of white Caucasian NIDDM-afflicted women, however, Zhang et al. (1996) reported that the prevalence of the IRS-1 972 mutation was significantly higher for NIDDM-subject groups featuring insulin resistance and dyslipidaemia than was the case for normal subjects (18 and 26% compared with 11% for the control subjects) [32]. We acknowledge that the subjects participating in our study were different to those participating in the study reported by Zhang et al, in that our study subjects included 47 patients featuring PCOS, of which there were 36.2% who revealed AGT, in addition to 45 normal women. Interestingly, we arrived at the same result as regards the absence of any codon 513 polymorphism as was the case for this abovementioned Japanese study, suggesting that this type of polymorphism is quite rare amongst the general Japanese and Taiwanese population. Nevertheless, it does appear that this study's Taiwanese test population reflected a lower incidence of polymorphism for codon 972 than was the case for the Japanese study, the polymorphism frequency figure being 3.6%/4.5% for the Japanese NIDDM/control groups and 0%/0% for our ninety-two subjects [31]. Further, comparison of the incidence of the IRS-1 972 mutation between white Caucasian, Japanese and Taiwanese subjects, revealed that the incidence was greatest amongst Caucasians (diseased/control: 26%/11%) [32], lower for Japanese subjects (3.6%/4.5%) [31], and quite rare for Taiwanese Hoklo and Hakka study subjects (0%/0%). In the phylogenetic tree, it would appear likely that the mainland Japanese revealed the closest affinity with continental Chinese and Korean people, these being principally Han Chinese [33,34]. Nevertheless, Taiwanese Hoklo and Hakka individuals, which comprised the majority of Taiwanese before the immigration of mainlanders, are closely related to southern Han Chinese people who appear to inherit haplotypes from both Han Chinese and Yueh individuals, making them genetically somewhat different from the northern Han Chinese and original Han Chinese people [17]. As for Caucasians, the Caucasoid is, in the genetic tree, more distant from the mainland Japanese, Hoklo, and Hakka [35]. Further evidence of genetic difference between different ethnic populations pertaining to the screening for PCOS or insulin resistance reveals the absence of any codon 972/513 polymorphism amongst Pima Indians of Arizona (USA), this rather well-defined sub-population featuring a high prevalence of NIDDM [36].

## Conclusion

Considering the statistical analysis on this scale of PCOS-affected subjects, being high risk of these polymorphisms, we cannot resoundingly declare a universal absence of codon 972/513 polymorphisms for Taiwanese PCOS women according to our study of 92 Taiwanese subjects. For this present study, however, we can conclude that no such polymorphism was found for all study subjects and that these polymorphisms were rare and less than other ethnic population. Since we have depicted the highly prevalence of AGT and insulin resistance in these PCOS affected patients, it suggests that these two IRS-1 polymorphisms are unrelated to the emergence of glucose dysmetabolism and insulin resistance in PCOS for the Taiwanese Hoko and Hakka population. We conclude that because the Taiwanese PCOS population does not include a sufficiently large number of cases of codon 972/513 mutation, these polymorphic alleles of IRS-1 are not a reliable or appropriate method of genetic diagnosis of PCOS featuring either AGT or insulin resistance, and that these polymorphic alleles of IRS-1 are quite rare amongst Taiwanese women.

## Abbreviations

AGT: abnormal glucose tolerance

Ala(A): alanine

Arg(R): arginine

Asp(D): aspartic acid

BMI: body mass index

bp: base pair

*Bst N1*: restriction endonuclease *BstN 1 *from Bacillus stearothermophilus Ndel: deletion

DNA: deoxyribonucleic acid

*Dra III *: restriction endonuclease *Dra III *from Deinococcus radiophilus

FSH: follicle stimulating hormone

G/I ratio: glucose/insulin ratio

Gly(G): glycine

HOMA: homeostasis model assessment index

HOMA_IR_: homeostasis model assessment index for insulin resistance

IRS: insulin receptor substrate

LH: luteinizing hormone

Met(M): methionine

NIDDM (non-insulin dependent diabetes mellitus)

PCO: polycystic ovary

PCOS: polycystic ovary syndrome

PCR: polymerase chain reaction

PCR-RFLP: polymerase chain reaction and restriction fragmented length

PI 3-kinase: Phosphatidylinostiol 3-kinase

polymorphism

Pro(P): proline

PTB: phosphotyrosine-binding

RFLP: restriction fragmented length polymorphism

Ser(S): serine

SH-2 domain: Src Homology-2 domain

SHBG: sex hormone binding globulinThr(T): threonine

Tyr(Y): tyrosine

YMXM motifs: Tyr-Met-X-Met motifs

## Competing interests

The author(s) declare that they have no competing interests.

## Authors' contributions

TCL conceived of the study, participated in its design, and drafted the manuscript. JMY performed the clinical coordinator and helped manuscript preparation. KBG performed the statistical analysis. TCK helped to plan the studies. DCK, SFL, and KBG carried out the DNA extraction and PCR-RFLP. MJW supervised and interpreted the studies of PCR-RFLP.

## Pre-publication history

The pre-publication history for this paper can be accessed here:


